# Virtual reality simulation for critical pediatric airway management training

**Published:** 2021-02-02

**Authors:** Elizabeth M. Putnam, Lauryn R. Rochlen, Erik Alderink, James Augé, Vitaliy Popov, Robert Levine, Alan R. Tait

**Affiliations:** ^1^Department of Anesthesiology, University of Michigan Health Systems, Ann Arbor, MI, USA; ^2^Department of Learning Health Sciences, University of Michigan Health Systems, Ann Arbor, MI, USA; ^3^Emergency Care Center, Jackson Memorial Hospital, Miami, FL and Archie MD, Inc., Boca Raton, FL, USA; ^4^Michigan Institute for Clinical and Health Research, University of Michigan Health Systems, Ann Arbor, MI, USA

**Keywords:** Virtual reality, simulation, pediatric airways, airway emergencies, medical education

## Abstract

**Background:**

Pediatric airway emergencies are relatively rare, but have potentially devastating consequences. Simulation based education is important in providing zero-risk management experience for these critical events.

**Aims:**

The aim of the study was to assess usability and feasibility of combined interactive instructional videos and a novel Virtual Reality (VR) trainer for healthcare professionals and to evaluate the impact of this combination on learners’ knowledge of critical airway events in children.

**Methods:**

The study population included medical students, residents, faculty, and advanced practice nurses. Participants completed a short baseline knowledge pre-test of pediatric airway emergency management, followed by these consecutive interventions: (1) Interactive instructional pediatric airway videos and (2) VR trainer (HoloLens technology), simulating a pediatric critical airway event. Participants were randomized to manage anaphylaxis or foreign body aspiration. Finally, participants completed a second knowledge test (post-test) and a survey of their perceptions of the videos and VR trainer.

**Results:**

Forty-one participants were included in the study. Overall, both interventions were well received. Positive perceptions included realism, interactivity, and active learning environment. Negative comments focused on video speed and the VR trainer learning curve. Participants reported preferences for future training of pediatric airway events to include videos and VR trainers, with or without didactic lectures. Most areas of knowledge showed slight to significant improvements following the interventions. Specifically, questions on pediatric anatomy, anaphylaxis, Heimlich maneuver, and foreign body removal showed the highest improvement in scores (*P* < 0.05).

**Conclusions:**

Interactive videos, in combination with a VR experience, provide promising zero-risk training for pediatric critical airway events.

**Relevance for Patients:**

Pediatric airway emergencies are relatively rare, but the potential consequences are devastating. VR is established as a valued mode of education with regard to medical emergency training. Multimedia informational and instructional formats result in greater understanding of information. Results from our intervention, combining an interactive video tutorial and a VR experience, show this was well received by a cross section of health-care providers. We demonstrated improved test scores in a pediatric airways quiz.

## 1. Introduction

The emergence of simulation-based models for medical education including mannequin training, virtual reality (VR), and interactive medical simulation software addresses the need for required skill development of health-care trainees [1]. Indeed, the relatively recent incorporation of high-tech, anatomically correct mannequins into medical education have shown great promise in terms of information comprehension, learner satisfaction, and patient outcomes [2-6].

The advantages of medical simulation training are seemingly obvious and well supported in the literature. It provides a safe environment for training in high-risk procedures, unlimited exposure to rare clinical events, the ability to reliably plan lessons, and the opportunity to repeat tasks [7]. However, the benefits of simulation programs are not always so clear cut. A weekly simulation-based pediatric intensive care unit training course was shown to improve 2^nd^-year pediatric residents’ comfort level, but not actual skill in performing intubations [8]. Trainees who underwent Neonatal Resuscitation Program simulation showed significant improvement in intubation skills immediately post-intervention, but this did not translate into improved clinical performance, which over time, returned to baseline [9]. These data suggest that improved performance in the simulation environment may not always transfer to the clinical setting. Furthermore, simulation is not widely accessible due to cost and availability of trained simulation faculty.

A promising approach to simulation for clinical procedural training is the use of VR. When compared to current mannequin–based simulation training, VR-based training offers several unique features that provide greater accessibility, the collection of dynamic behavioral data, and increased immersion and realism. This provides opportunities for training healthcare workers on critical “high acuity, low frequency” events that are difficult to recreate in real life and allows them to make decisions, and mistakes, without risk to the patient [10]. VR technology has been used in pediatrics as a distraction tool, and to prepare children for hospital experiences [11], and in specific skill training for fiber optic laryngoscopy [12]. There has been minimal research using immersive VR as training tool for learners, with regard to diagnosis and management of pediatric airway emergencies. VR typically involves the use of headsets or goggles (e.g., HoloLens^®^ technology, Microsoft Corp., Redmond, WA) to place the user’s physical presence into a virtual or imaginary setting. Within these virtual environments the user can interact with visual characters (avatars) in different scenarios. Visual perception plays a key role in learning [12]; it helps students understand complex processes by converting an abstract concept into specific visual objects and events. Well-designed visual tools allow students to rapidly absorb complex materials [13,14]. Furthermore, high-fidelity avatars have the ability to express emotions or conditions facilitating a sense of reality in a virtual world. VR training allows for all virtual interactions to be recorded and reviewed for debriefing and teaching purposes. Importantly, whereas conventional simulation training provides a more varied experience between learners, VR can offer a more consistent learning experience by providing stimuli that are standardized and respond to learners’ responses in a more reliable manner.

Pediatric and neonatal emergency airway management skills are life-saving, difficult to master, and used infrequently enough to make skill acquisition a challenge. For example, a number of studies have documented low pediatric and neonatal intubation success rates. In one study, only 44% of 455 neonatal tracheal intubation attempts were successful with success rates ranging from 72.2% among attending physicians to only 20.3% among pediatric residents [15]. A study at the University of Texas Health Science Center found that 35% (160 of 449) of intubation procedures were never successfully completed by pediatric residents [16]. Difficulties in the ability to visualize the vocal chords and the position of the laryngoscope tip in the vallecular and under the proximal epiglottis have been cited as reasons for failed intubation [15,17].

Pediatric airway emergencies are relatively rare but the potential consequences dire, thus simulation training is critical in providing exposure to, and experience with, managing these important events. This study, therefore, was designed to evaluate the use of interactive instructional videos in combination with a novel VR trainer as a means to train health-care professionals on appropriate airway management techniques for various pediatric airway emergencies.

## 2. Materials and Methods

This study was deemed exempt by the university’s Institutional Review Board.

### 2.1. Product development

#### 2.1.1. Interactive instructional videos

**Figure 1:**
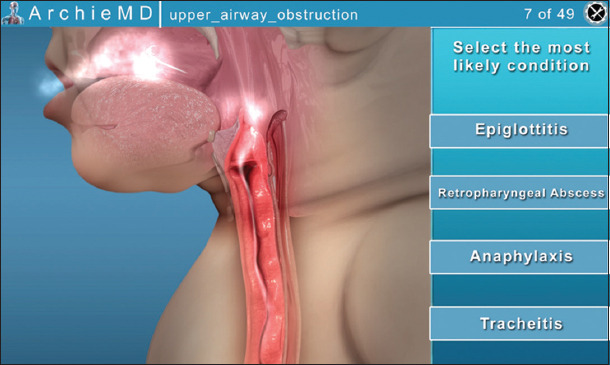
Image 1: Excerpt from the instructional video interactive quiz.

The initial content for the instructional videos was generated using the extant literature, expert opinion, and iterative review by board-certified anesthesiologists (pediatric and adult) and an emergency room physician. Scripts were developed and three-dimensional images designed by graphic artists using several software programs including Maya, 3D Studio Max, Adobe After Effects, Adobe Photoshop, and Macromedia Flash. HTML and JAVA were employed. Once the content and the visual models/simulations were created they were merged to form individual anatomical and event-specific video modules, that is pediatric airway anatomy, anaphylaxis/angioedema, epiglottitis, foreign body aspiration, infant croup, and pediatric burn. The total duration was 45 min, and at the conclusion of each module, an interactive quiz tested real-time understanding of the information. Feedback was given after each quiz Figure 1.

#### 2.1.2. VR program

**Figure 2:**
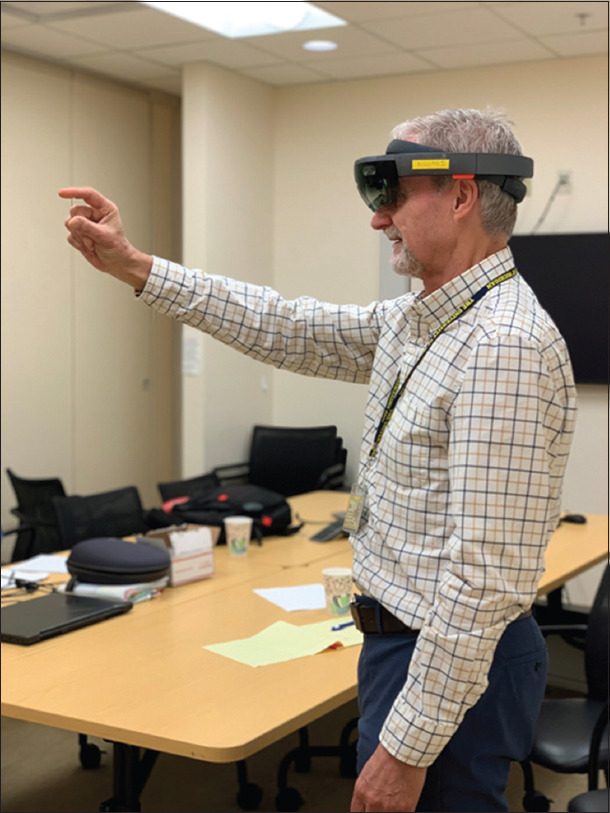
Image 2: Practicing directive gestures using the virtual reality trainer.^1^

The content for the VR program was developed using the same iterative approach. Three-dimensional visual models/simulations were generated using similar software to that used in the videos, but to provide a VR experience the 3D visuals were rendered using unity game theory and scaled and registered using Art Toolkit. Event-specific modules were then created to mirror those of the videos, for example, pediatric epiglottitis, and anaphylaxis and viewed by the user in the virtual world using HoloLens^®^ technology (Microsoft Corp., Redmond, WA). This allowed the user to immerse themselves in any of the virtual pediatric medical scenarios with the opportunity to make decisions and “manage the case Figure 2.”

Two clinical scenarios (foreign body aspiration and anaphylaxis/angioedema) were selected for usability testing. Multiple interventions and management options were available to the participants during the scenario. Only correct interventions, registered with the system in the correct sequence, and allowed the participant to progress through the scenario.

#### 2.1.3. Usability testing of the VR program

A prototype of the VR program was tested for usability by ten health-care individuals with varying degrees of pediatric airway management experience (none to highly experienced). Participants adopted a “talk back” approach to elicit feedback on their experience. Comments were documented and relayed back to the development team for review and refinement.

#### 2.1.4. Product evaluation

The study population included first through 4^th^-year medical students attending this institution’s Medical School together with residents, fellows, faculty, nurses, advanced practice nurses, and nurse anesthetists (CRNAs) from the Departments of Anesthesiology, Pediatric Critical Care, Pediatrics, and Emergency Medicine. Participants were identified through the University of Michigan’s e-mail listings and face-to-face recruitment.

Participants were shown the interactive instructional videos which provided information on pediatric airway anatomy and the diagnosis and management of the aforementioned critical airway presentations. This was followed with the VR trainer and a brief orientation to the HoloLens’^®^ directive gestures (Figure 2). Participants were randomized (by alternate day testing) to manage one of two airway scenarios (anaphylaxis/angioedema or foreign body aspiration). Each participant was allowed up to three attempts to manage the patient.

Each participant action (decision) was recorded in the VR system and decisions were reinforced by real-time visual and sound feedback (through the VR program) indicating a correct or incorrect action. To test usability, each participant completed an online survey (Qualtrics,^®^ Provo, Utah) to elicit information about their level of training, experience with pediatric airway management, and perceptions of the interactive videos and VR technology. All participants completed the same short quiz, before and immediately following the educational intervention. This comprised 19 true/false questions focused on the content of the educational videos and specific VR scenarios. This quiz was designed by the authors and covered baseline knowledge of pediatric airway anatomy and complications, and specific “new” knowledge. This reflected both the broad and specific material covered in the educational videos, and the management of anaphylaxis and an aspirated foreign body experienced in VR trainer. The true/false format was chosen for the benefits of being a brief, simple, closed end format, and familiar to participants.

### 2.2. Statistical analysis

Sample size determination was based on a convenience sample. Since we anticipated that most participants would have limited prior education and knowledge of pediatric airway management, we believed that a 50% increase in understanding would be attainable following exposure to the instructional videos and the VR trainer. This sample was sufficient to detect a difference of at least that size (b = 0.2, two-tailed)). Qualtrics^®^ and HoloLens^®^ data were downloaded to SPSS version 25.0 (IBM Inc., New York, NY) for analysis. Quantitative data were analyzed using descriptive statistics. Non-parametric analyses were performed using Wilcoxon and McNemar’s tests. Data are median and n (%). *P* < 0.05 was considered statistically significant. Free-text responses to open-ended questions were evaluated using inductive thematic analysis [18] and the most common themes reported.

## 3. Results

Table 1 describes the demographics of 41 participants. As shown, there was a wide range of experiences and training.

**Table 1:** Demographics

	*n* (%)
Gender (F/M) %	51.2/48.8
Level of training	
Medical Student	8 (19.5)
Resident	6 (14.6)
Fellow	3 (7.3)
Nurse (Nurse/Nurse Practitioner/CRNA)	10 (24.5)
Faculty	14 (34.1)
Specialty	
Anesthesiology	14 (37.8)
Emergency Medicine	11 (26.8)
Pediatric Critical Care	6 (16.2)
General Pediatrics	2 (5.4)
Other	4 (10.8)
Prior experience	
Prior training/education in pediatric airway management	32 (78.0)
Pediatric airway training with simulator	16 (50.0)
Number of past cases requiring pediatric airway management	5 (12.2)
None	11 (26.8)
1-10	6 (14.6)
11-40>40	19 (46.3)

CRNA: Certified registered nurse anesthetist

Table 2 describes the participants’ perceptions of the instructional videos. These results showed very positive overall impressions.

**Table 2:** Participants’ perceptions of the instructional videosa

	SD	D	Neither	A	SA
Airway scenarios were realistic	0 (0.0)	0 (0.0)	0 (0.0)	20 (48.8)	21 (51.2)
Pediatric anatomy was realistic	0 (0.0)	0 (0.0)	1 (2.4)	12 (29.3)	28 (68.3)
Information was comprehensive	0 (0.0)	1 (2.4)	0 (0.0)	16 (39.0)	24 (58.6)
Features supported learning	0 (0.0)	0 (0.0)	0 (0.0)	10 (24.4)	31 (75.6)
Improved my knowledge	0 (0.0)	1 (2.4)	2 (4.9)	11 (26.8)	27 (65.9)
Improved my confidence	0 (0.0)	2 (4.9)	4 (9.8)	20 (48.8)	15 (36.6)
Interactive quizzes were helpful	0 (0.0)	0 (0.0)	1 (2.4)	12 (29.3)	28 (68.3)

SD: Strongly Disagree; D: Disagree; Neither: Neither disagree nor agree; A: Agree; SA: Strongly agree; Data are *n* (%)

Open-ended comments from participants regarding their perceptions of the instructional videos were overall very positive. The majority of these comments related to the realism of the videos. The following comments are taken verbatim:

*“The dynamic nature of the videos was good. Interesting to see the anatomy immediately change in front of you. Coupling the sight with the sound change was helpful too.”*

*“I liked that the anatomy was clearly shown. I like that the changes expected for each pathology were also clearly shown visually. I also liked having the questions after each section to confirm understanding/make it more interactive/active.”*

*“The visuals and audios that accompanied the videos were helpful for both keeping me engaged and providing realistic visuals to anchor the information presented. They gave a great overview.”*

Dislikes were minimal and focused on the video narration pace.

*“I would have liked to be able to listen a little faster. The pace of talking was a little slow. Maybe allow for people to increase the speed if they desire?”*

*“Pace of the voice was pretty slow, and while the robotic pronunciations weren’t a deal breaker, they were somewhat distracting. Maybe a little too simple depending on skill level.”*

*“Animations were admittedly a bit creepy and unnerving (not your fault though!) The follow-up questions* (embedded within the videos) *were a bit too basic and didn’t really challenge learning, nor reinforce concepts when questions were wrong. Perhaps a follow-up section which reviews concepts after wrong questions would help?”*

Table 3 describes the participants’ perceptions of the VR trainer on a 5-point Likert scale ranging from strongly disagrees to strongly agree.

**Table 3:** Participants’ perceptions of the virtual reality trainer

	SD	D	Neither	A	SA
VR anatomy was realistic	2 (4.9)	3 (7.3)	3 (7.3)	27 (65.9)	6 (14.6)
Ability to see internal structures	0 (0.0)	3 (7.3)	13 (31.7)	12 (29.3)	12 (31.7)
Real-time feedback was helpful	3 (7.3)	3 (7.3)	5 (12.2)	17 (41.5)	13 (31.7)
VR trainer was easy to use	3 (7.3)	4 (9.8)	9 (22.0)	23 (56.1)	2 (4.9)
VR trainer was enjoyable	2 (4.9)	1 (2.4)	5 (12.2)	13 31.7)	20 (48.8)
VR trainer improved my confidence	4 (9.8)	6 (14.6)	11 (26.8)	11 (26.8)	9 (22.0)
VR trainer promoted learning	2 (4.9)	3 (7.3)	2 (4.9)	13 (31.7)	21 (51.2)
VR useful for skills training	0 (0.0)	1 (2.4)	3 (7.3)	12 (29.3)	25 (61.0)
Incorporate VR into medical training	0 (0.0)	2 (4.9)	3 (7.3)	16 (39.0)	20 (48.8)

SD: Strongly disagree; D: Disagree; Neither: Neither disagree nor agree; A: Agree; SA: Strongly agree; Data are *n* (%)

Again, open-ended responses to the VR trainer were generally positive. Likes were mostly related to realism, ability to actively practice learned knowledge and skills, and value of immediate feedback.

*“I liked that I could interact with the patient and perform physical exam maneuvers. I liked that it would give times to understand how the patient responded to treatment.”*

*“It helped to create a more active learning environment to apply what I had just seen in the videos. This helped me to integrate/think on my feet about the knowledge from the videos and also identified areas that I needed to restudy before trying again. Being able to look into the mouth of the virtual baby and see a foreign body helped to solidify my next steps (rather than just talking about what to in a lecture setting).”*

*“Ease of use, incorporation of feedback into scenario, much more interactive than sitting in front of a computer, ability to have many different management choices and also operate under time pressure.”*

Dislikes of the VR program were mostly related to unfamiliarity with the technology and HoloLens gestures.

*“I think with more practice, the gestures you make to interact with the simulation will become more second nature. It was a little bit clunky in terms of timing for when you were allowed to use certain instruments.”*

*“The options available were not well set out to see. Vitals should be running in a corner of the screen as they would in a patient room. Need age of patient if we are going to perform age related interventions.”*

*“No intubation practice on the VR technology, clunky interface that was difficult to interact with, incomplete medical management of a simple case (lacked the use of other medication adjuncts, ability to see monitor for vitals, no discussion of pt. disposition).”*

Following exposure to the instructional videos, participants were asked to rate their confidence in managing the airway scenario presented to them in the VR trainer and how helpful the videos were in preparing them. Results showed that confidence was rated as 6.73 ± 3.00 (0-10 scale where 10 = Extremely confident). Helpfulness of the instructional videos in the participants’ ability to diagnose and manage the scenario presented in the VR trainer was rated as 7.93 ± 1.99 and 7.27 ± 2.08, respectively (0-10 scale where 10 = Extremely helpful). Participants were also asked to rank their preference for training in critical pediatric airway management, that is, didactic lectures only; instructional videos only; VR trainer only; both instructional videos and VR trainer; a combination of lectures, videos and VR trainer; and finally, none of these options. Median ranks (where lower ranks signify increasing preference) were 4.0, 3.0, 4.0, 2.0, 2.0, and 6.00, respectively.

Table 4 describes the results from the pre- and post-tests. This table shows improvement in knowledge in several questions. These results were consistent when stratified by level of training, that is, trainees (medical and nursing students, residents) and established practitioners (CRNAs, nurse practitioners, fellows, and faculty).

**Table 4:** Pre-versus post-test: correct responses – All participants (%)

	Pre	Post	*P* value
When intubating an infant, a rolled blanket should be placed under the occiput to help with aligning the oral, pharyngeal and tracheal axis, for an optimal position. (False)	63.4	73.2	0.366
Compared to the adult larynx, the infant larynx is more anterior. (True)	90.0	100.0	0.046
Edema and swelling in croup is restricted to the oropharynx. (False)	100.0	95.1	0.157
In a child, laryngospasm will only partially obstruct the airway. (False)	95.0	97.6	0.564
A nasal pharyngeal airway (NPA) can bypass tongue swelling to improve ventilation in a child. (True)	90.0	97.6	0.083
For a child with a partially obstructed airway, an oral airway adjunct is an appropriate initial choice when the child is awake. (False)	90.0	97.6	0.180
In a partial airway obstruction of a child, intubation should be performed immediately. (False)	85.0	95.1	0.157
Children presenting with anaphylaxis typically will have a rash. (True)	35.0	92.7	0.000
In anaphylaxis the first line of treatment is oxygen and epinephrine. (True)	95.0	100.0	0.317
In anaphylaxis, once epinephrine has been given, a second dose should not be given. (False)	97.5	100.0	0.317
H2 blockers such as ranitidine can be used in the management of anaphylaxis. (True)	85.0	92.7	0.317
You are presented with a toddler who has possibly aspirated a foreign body. The presence of coughing and crying is suggestive of a complete airway obstruction. (False)	100.0	100.0	1.00
The Heimlich maneuver is appropriate to use once children are over 1 year of age. (True)	35.0	92.7	0.000
To remove a foreign body which is visible above the vocal cords, the McGill forceps should be inserted into the mouth in the open position. (False)	52.5	80.5	0.001
The incidence of foreign body aspiration is highest in infants under 1 year. (False)	85.0	87.8	0.480
Following a house fire, smoke inhalation often causes anaphylaxis. (False)	90.0	90.2	1.00
A child with airway burns may present with stridor or wheezing or both. (True)	100.0	100.0	1.00
Early intubation is the treatment for a child with airway burns and signs of airway edema. (True)	97.6	100.0	0.317
Following trauma, cervical spine precautions do not need to be taken in children during intubation. (False)	100.0	95.1	0.157

Responses to questions are True/False (correct answers given). Results indicate the % of correct responses.

## 4. Discussion

Results of this study showed that a combination of interactive instructional videos and a novel VR trainer was well received and deemed usable, by providers with a wide range of experiences, in learning about the management of several important critical airway events seen in children. Overall, the instructional videos and VR trainer were better received than the VR trainer although VR also received a preponderance of positive reviews. However, participants’ ranking suggests that a combination of videos and the VR trainer with or without didactic lectures were the preferred method of instruction for the understanding and management of pediatric critical airway events. The participants enjoyed the interactive videos which provided narration, realistic images, and audio. Several participants, in particular, remarked on the realism of the sound effects associated with airway compromise (e.g., stridor and wheeze) to the extent that they actually evoked a degree of anxiety commensurate with the real event. The interactivity of the videos and embedded quizzes was well received. Several studies have shown that highly visual interactive modalities help individuals assimilate and understand information better because they provide greater visual saliency (pictorial superiority effect), and promote active participation in learning rather than the more traditional passive reading of text [19-21]. The VR trainer was well received in terms of educational value although enthusiasm, for some, was dampened by technological glitches and unfamiliarity with the technology. While these glitches were a distraction for some, they are consistent with the development of any prototype and were all deemed fixable.

Although some areas of knowledge were not improved following the interventions, most participants showed small to significant improvements in their understanding of pediatric anatomy and emergency airway management, particularly in specific areas addressed by the videos and VR experience. These included proper use of the Heimlich maneuver, correct use of the Magill forceps, and physical exam findings in anaphylaxis. That all areas of instruction were not improved significantly may reflect the nature of some of the questions (i.e., too simple) and/or that many of the participants were relatively experienced and, as such, would not be expected to show substantial increases in knowledge. It could also be that despite significant improvements in understanding in some areas, the study may have been under-powered to detect significant differences in all. There is opportunity to better study our intervention’s impact on knowledge, using a more extensive assessment tool, longer follow-up, a control group, and a larger study population.

The potential limitations of this study are acknowledged. First, the study evaluates one VR prototype at one institution and thus may not be generalizable to all institutions and populations. Although our initial thought was to focus primarily on trainees such as medical students, we were pleasantly surprised in the interest generated from a broad range of specialties and training levels. Although including participants with prior clinical experience may have reduced the impact of the interventions on knowledge acquisition, this allowed us to gain a broader perspective on the overall impact that this approach might have on both educators and trainees. Technical acquisition of skills such as pediatric intubation was not assessed in this study; future VR work perhaps employing haptic technology could usefully investigate this. In addition, although an outcome measure demonstrated increased confidence in management in pediatric airways, the authors acknowledge that an increase in confidence is only helpful with an increase in technical or decision making skills. An additional limitation is the brief true/false questions for the knowledge test. Incorporating a more detailed knowledge assessment, such as multiple choice questions, could provide more robust data on the knowledge gaps and learning of the participants.

Although initial outlay for a VR headset can be expensive, there is a long-term cost-benefit in the ability of VR to improve accessibility of a large number of trainees to simulation training with minimal ongoing costs.

Of interest, was the observation that a majority of participants reported a desire for this type of combined approach (interactive videos and VR trainer) for the education of both trainees and trained personnel. Previous work has shown that multimedia informational and instructional formats result in greater understanding of information [22,23]. This likely occurs because the immersive, interactive and novel aspects of multimedia learning, including VR, promote active participation and engagement.

## 5. Conclusion

Although many of the critical airway events that occur in children are not particularly common, they are potentially catastrophic. Since clinical exposure to these events may be limited, exploration of other types of exposures using simulation and virtual technology is important. To the best of our knowledge, this study is the first to apply VR technology to the training of critical airway management in children. Given the increasing emphasis on alternative education modalities, this combination of highly immersive and interactive videos with a VR experience, appears promising as a means to enrich education.

## Footnotes

1 The person in this picture has consented to being displayed in this article and does not consider this a violation of his privacy

## References

[1] Hao J, Estrada J, Tropez-Sims S (2002). The Clinical Skills Laboratory:A Cost-Effective Venue for Teaching Clinical Skills to Third-Year Medical Students. Acad Med.

[2] Weller J (2004). Simulation in Undergraduate Medical Education:Bridging the Gap Between Theory and Practice. Med Educ.

[3] Block EF, Lottenberg L, Flint L, Jakobsen J, Liebnitzky D (2002). Use of a Human Patient Simulator for the Advanced Trauma Life Support Course. Am Surg.

[4] Issenberg S, McGaghie WC, Hart IR, Mayer JW, Felner JM, Petrusa ER (1999). Simulation Technology for Health Care Professional Skills Training and Assessment. J Am Med Assoc.

[5] Gorden J, Oriol N, Cooper J (2004). Bringing Good Teaching Cases “To Life”:A Simulator-Based Medical Education Service. Acad Med.

[6] Flanagan B, Nestel D, Joseph M (2004). Making Patient Safety the Focus:Crisis Resource Management in the Undergraduate Curriculum. Med Educ.

[7] Grenvik A, Schaefer JJ, DeVita MA, Rogers P (2004). New Aspects on Critical Care Medicine Training. Curr Opin Crit Care.

[8] Tofil NM, Benner KW, Zinkan L, Alten J, Varisco BM, White ML (2011). Pediatric Intensive Care Simulation Course:A New Paradigm in Teaching. J Grad Med Educ.

[9] Patel J, Posencheg M, Ades A (2012). Proficiency and Retention of Neonatal Resuscitation Skills by Pediatric Residents. Pediatrics.

[10] Bracq MS, Michinov E, Jannin P (2019). Virtual Reality Simulation in Nontechnical Skills Training for Healthcare Professionals:A Systematic Review. Simul Healthc.

[11] Evans C, Moonesinghe R (2020). Virtual Reality in Pediatric Anesthesia:A Toy or a Tool. Paediatr Anaesth.

[12] Sekuler R, Blake R (1985). Perception.

[13] Kraidy U (2002). Digital Media and Education:Cognitive Impact of Information Visualization. J Educ Med.

[14] Linn M, Songer N, Eylon B, Calfee R, Berliner D (1996). Shifts and convergences in science learning and instruction. Handbook of Educational Psychology.

[15] Haubner LY, Barry JS, Johnston LC, Soghier L, Tatum PM, Kessler D (2013). Neonatal Intubation Performance:Room for Improvement in Tertiary Neonatal Intensive Care Units. Resuscitation.

[16] Falck AJ, Escobedo MB, Baillargeon JG, Villard LG, Gunkel JH (2003). Proficiency of Pediatric Residents in Performing Neonatal Endotracheal Intubation. Pediatrics.

[17] Donoghue A, Ades A, Nishisaki A, Zhao H, Deutsch E (2013). Assessment of Technique during Pediatric Direct Laryngoscopy and Tracheal Intubation:A Simulation-Based Study. Pediatr Emerg Care.

[18] Saldaña J (2015). The Coding Manual for Qualitative Researchers.

[19] Ally B, Budson A (2007). The Worth of Pictures:Using High Density Event-Related Potentials to Understand the Memorial Power of Pictures and the Dynamics of Recognition Memory. Neuroimage.

[20] Cherry K, Park D, Frieske D, Smith AD (1996). Verbal and Pictorial Elaborations Enhance Memory in Younger and Older Adults. Aging Neuropsychol Cogn.

[21] Hockley W (2008). The Picture Superiority Effect in Associative Recognition. Mem Cogn.

[22] Tait AR, Voepel-Lewis T, Moscucci M, Brennan-Martinez C, Levine R (2009). Patient Comprehension of an Interactive, Computer-Based Information Program for Cardiac Catheterization:A Comparison with Standard Information. Arch Intern Med.

[23] Hermann M (2002). 3-Dimensional Computer Animation--A New Medium for Supporting Patient Education Before Surgery. Acceptance and Assessment of Patients Based on Prospective Randomized Study--Picture Versus Text. Chirurg.

